# Associations of apolipoprotein E ε4 allele, regional cerebral blood flow, and serum liver function markers in patients with cognitive impairment

**DOI:** 10.3389/fneur.2024.1345705

**Published:** 2024-04-02

**Authors:** Hao Wang, Lin Shi, Shimei Luo, Yishan Luo, Chunyan Xu, Guozhen Qiu, Qiwen Guo, Chunchun Chen, Taikun Lu, Kangding Liu, Feiqi Zhu

**Affiliations:** ^1^Department of Neurology, The First Hospital of Jilin University, Changchun, Jilin, China; ^2^BrainNow Research Institute, Guangdong, China; ^3^Department of Nuclear Magnetic Resonance, The Third Affiliated Hospital of Shenzhen University Medical College, Shenzhen, China; ^4^Cognitive Impairment Ward of Neurology Department, The Third Affiliated Hospital of Shenzhen University Medical College, Shenzhen, China

**Keywords:** Alzheimer’s disease, amnestic mild cognitive impairment, apolipoprotein E ε4 allele, cerebral blood flow, liver function marker, arterial spin labeling

## Abstract

**Introduction:**

The ε4 allele of the apolipoprotein E gene (APOE4) is expressed abundantly in both the brain and peripheral circulation as a genetic risk factor for Alzheimer’s disease (AD). Cerebral blood flow (CBF) dysfunction is an essential feature of AD, and the liver plays an important role in the pathogenesis of dementia. However, the associations of APOE4 with CBF and liver function markers in patients with cognitive impairment remains unclear. We aimed to evaluate the associations of APOE4 with CBF measured by arterial spin labeling (ASL) magnetic resonance imaging (MRI) and serum liver function markers in participants who were diagnosed with cognitive impairment.

**Methods:**

Fourteen participants with AD and sixteen with amnestic mild cognitive impairment (MCI) were recruited. In addition to providing comprehensive clinical information, all patients underwent laboratory tests and MRI. All participants were divided into carriers and noncarriers of the ε4 allele, and T-tests and Mann–Whitney U tests were used to observe the differences between APOE4 carriers and noncarriers in CBF and liver function markers.

**Results:**

Regarding regional cerebral blood flow (rCBF), APOE4 carriers showed hyperperfusion in the bilateral occipital cortex, bilateral thalamus, and left precuneus and hypoperfusion in the right lateral temporal cortex when compared with noncarriers. Regarding serum liver function markers, bilirubin levels (including total, direct, and indirect) were lower in APOE4 carriers than in noncarriers.

**Conclusion:**

APOE4 exerts a strong effect on CBF dysfunction by inheritance, representing a risk factor for AD. APOE4 may be related to bilirubin metabolism, potentially providing specific neural targets for the diagnosis and treatment of AD.

## 1 Introduction

Alzheimer’s disease (AD) is a progressive neurodegenerative disorder and the most common type of dementia, imposing a large burden on our increasingly aging population (1). The hallmark of AD pathogenesis is the accumulation and deposition of amyloid-β (Aβ) plaques in the brain (2). Amnestic mild cognitive impairment (MCI) is identified as a prodromal phase of AD, with approximately 10–20% of MCI patients progressing to dementia annually (3). Nevertheless, the prevalence of dementia continues to rise while therapeutic options remain limited, urging more researchers to target modifiable risk factors and develop effective strategies for prevention and treatment.

The ε4 allele of the apolipoprotein E gene (APOE4) is considered the strongest genetic risk factor for AD, increasing the risk of AD progression and decreasing the age of onset in a gene dose-dependent manner (4, 5). APOE4 not only leads to earlier and higher Aβ concentration but also affects non-Aβ pathways such as vascular function, immune responses, and tau-mediated neurodegeneration (6). APOE4 can also accelerate the progression of age-related cognitive impairment in individuals without dementia (7). However, specific associations between APOE4 and AD are still poorly understood because of the complexity of the underlying pathophysiological mechanisms (8).

APOE is primarily generated by astrocytes in the central nervous system (CNS) and more than 90% of peripheral APOE in the plasma is produced by the liver, and it plays a major role in numerous biological processes in both areas (9, 10). In the CNS, multiple mechanistic pathways regulated by APOE are collectively associated with cognitive function (11). As a crucial lipid carrier, APOE participates in supporting membrane homeostasis, synaptic integrity, and injury repair in the brain. In peripheral circulation, APOE modulates lipid-related metabolism through its release into the blood (6). A recent review summarized APOE4’s key role in cerebrovascular dysfunction through various mechanisms, including cerebral blood flow (CBF) disruption, cerebral amyloid angiopathy with slower Aβ clearance, and blood–brain barrier leakage, and these detrimental changes may promote cognitive decline (11). Many studies have indicated that APOE4 carriers exhibit abnormal CBF in certain brain regions when compared to noncarriers (11). However, the associations of APOE4 with CBF in cognitively impaired patients have been inconsistent and inconclusive in previous studies.

Interestingly, peripheral APOE might also affect cerebrovascular and cognition despite physical separation (12). Liu et al. demonstrated that accumulation of peripheral APOE4 was sufficient to damage brain function and exacerbate amyloid pathogenesis in mouse models (6). Since peripheral APOE4 primarily originates from the liver, a growing body of evidence suggests that the liver may also play an important role in the pathogenesis of dementia. Taken together, we propose that a complex interaction exists between APOE4 and liver function markers.

Therefore, we aimed to explore whether APOE4 associates with brain perfusion and to assess the potential association between APOE4 and liver function marker levels in patients with cognitive impairment.

## 2 Methods

### 2.1 Participants

From 2019 to 2022, 88 participants with primary complaints of memory decline were recruited from the Outpatients Department of Cognitive Disorders at the geriatric branch of Luohu District People’s Hospital in Shenzhen. This study was approved by the Medical Research Ethics Committee of The Third Affiliated Hospital of Shenzhen University Medical College. All participants with memory loss were evaluated by clinicians specializing in dementia disorders, who also recorded clinical histories, physical examinations, and neurological tests. Additionally, they underwent laboratory tests, MRI scans, and standard neuropsychological assessments. Participants were excluded if they had been diagnosed with any of the following conditions: (1) other neurological diseases such as vascular dementia, Lewy body dementia, Parkinson’s disease dementia, progressive supranuclear palsy, multiple system atrophy, mixed dementia, or lues nervosa; (2) mild or severe liver diseases such as fatty liver, viral hepatitis, or hepatocirrhosis; (3) acute illness such as acute infectious diseases or severe mental disorder such as schizophrenia; (4) abuse of alcohol or psychoactive substances; or (5) other diseases that may interfere with cognitive evaluations and serum liver function marker levels. Based on the inclusion and exclusion criteria, 34 participants were retained for the current study.

### 2.2 Diagnostic criteria for Alzheimer’s disease and amnestic mild cognitive impairment

Lumbar puncture was performed in 34 participants, and the Aβ (1–40), Aβ (1–42), phosphorylated tau protein (181), and total tau protein in cerebrospinal fluid were tested with the ELISA technique at Omeng Weiyi Medical laboratory in Hangzhou, according to diagnostic guidelines provided by the National Institute on Aging and Alzheimer’s Association (NIA-AA) (13). All AD patients met the following criteria: (1) Aβ (1–40) <550 pg./mL; (2) Aβ1-42/Aβ1-40 ≤ 0.1; (3) phosphorylated tau protein (181) >61 pg./mL; (4) total tau protein >452 pg./mL. Based on recommendations from NIA-AA workgroups (14), MCI patients were diagnosed if they met the criteria for the clinical and cognitive syndrome: (1) concern regarding a change in cognition; (2) impairment in one or more cognitive domains; (3) preservation of independence in functional abilities; and (4) not demented.

### 2.3 MRI data acquisition and cerebral blood flow analysis

Arterial Spin Labeling (ASL) MRI is a noninvasive method for measuring perfusion. ASL MRIs (TR: 4 s; TE: 16.36 ms; slice thickness: 3 mm; spacing: 3.28 × 3.28 mm; matrix: 64 × 63) were processed and analyzed using AccuBrain (BrainNow Medical Technology Ltd., Hong Kong, Hong Kong SAR). With this technique, it is possible to create a perfusion-weighted image related to the CBF image that reflects each voxel’s quantitative perfusion by subtracting the label and control image. Meanwhile, the subject’s T1-weighted imaging was segmented to obtain automated anatomical atlas (AAL) (15) labels. The AAL labels were transformed into an individual’s CBF image space via rigid registration. The absolute and standardized CBF values (normalized by the personal mean CBF value) in different regions were calculated and analyzed, included the left and right parts of the temporal, parietal, occipital, orbitofrontal, frontal, and sensorimotor cortices, as well as the precuneus, gyrus rectus, anterior and posterior cingulate, thalamus, and cerebellum.

### 2.4 Serum liver function markers

All participants fasted overnight and were tested at 8 a.m. the following morning. Each subject provided 3 mL of peripheral venous blood, and fresh serum was immediately used to analyze liver function markers including total protein (TP), albumin (ALB), globin (GLB), total bilirubin (TB), direct bilirubin (DB), indirect bilirubin (IB), alanine transaminase (ALT), aspartate aminotransferase (AST), γ-glutamyl transpeptidase (GGT), and alkaline phosphatase (ALP). With this combination of indicators, we were also able to calculate the ratio of albumin to globin (A/G) and the ratio of aspartate aminotransferase to alanine transaminase (AST/ALT) for further analysis.

### 2.5 Apolipoprotein E genotyping

Venous blood (2 mL) and DNA were extracted from 30 participants (14 participants with AD and 16 with MCI) using a blood nucleic acid extraction kit (Selechi Biotechnology Ltd., Zhuhai, China). Four participants did not volunteer due to economic reasons. Amplification was performed by quantitative real-time polymerase chain reaction (qRT-PCR) and hybridized with specific nucleic acid probes on the gene chips (Selechi Biotechnology Ltd.) to determine the APOE gene locus sequence. Thirty participants were tested for the APOE genotype and then classified into two groups: 13 carriers (APOE4+) and 17 noncarriers (APOE4−) of the ε4 allele. To investigate the impact of APOE4 in patients with cognitive impairment, we integrated the AD and MCI groups as a pooled group of cognitively impaired participants.

### 2.6 Neuropsychological tests

To assess the stage of cognitive decline, all participants completed the Mini-Mental State Examination (MMSE) and 22 finished Montreal Cognitive Assessment (MoCA) tests that had been adapted into a Chinese version. For various reasons (e.g., the patient’s cognitive decline was so severe that they were unable to finish senior testing), two MoCA scores were unavailable for the APOE4+ and six were unavailable for the APOE4− group.

### 2.7 Statistical analysis

IBM SPSS Statistics, version 25.0 (IBM Corp., Armonk, NY, United States) was used to conduct the following statistical analyses. For demographic characteristics, continuous variables are presented as mean ± standard deviation (SD), whereas categorical variables are expressed as frequencies (%). To describe general characteristic differences between the APOE4+ and APOE4− groups, t-tests were used to examine continuous variables (e.g., age, education years, MMSE scores) and chi-square tests were used to examine categorical variables (e.g., sex and hypertension). We used Fisher’s exact test to measure categorical variables because the total sample capacity was less than 40. To show differences in regional cerebral blood flow (rCBF) and liver function marker levels between APOE4 carriers and noncarriers, we used t-tests when two sets of data conformed to normal distribution, otherwise, the Mann–Whitney U-tests were used. These statistical tests were reported with a 95% confidence interval (CI). Differences in rCBF and serum liver function markers between the two groups were examined using a two-tailed t-test or Mann–Whitney U-test. The two-tailed significance threshold was set at *p* < 0.05.

## 3 Results

### 3.1 Sample characteristics

For general demographic characteristics (Table 1), the APOE4+ group did not differ significantly from the APOE4− group in sex, age, education years, hypertension, diabetes mellitus, hyperlipidemia, heart disease, and family history of dementia (*p* > 0.05). Additionally, there were no significant neuropsychological differences between the two groups, as assessed by the MMSE and MoCA scores (*p* > 0.05). After genotype classification, the constituent ratios of the types of dementia were also not statistically different (*p* > 0.05; Table 2).

**Table 1:** Demographic and clinical characteristics of participants.

Variables		APOE4+ (*n* = 13)	APOE4− (*n* = 17)	t value	*p* value
Sex, n (%)	male	2 (15.4)	8 (47.1)		0.119	female
		11 (84.6)	9 (52.9)		
Age, years		66.23 ± 8.60	68.65 ± 10.78	−0.662	0.513
Education years, years		10.83 ± 3.13	10.64 ± 5.30	0.113	0.911
Hypertension, n (%)		7 (53.8)	7 (41.2)		0.713
Diabetes mellitus, n (%)		3 (23.1)	4 (23.5)		1.000
Hyperlipoidemia, n (%)		9 (69.2)	10 (58.8)		0.708
Heart disease, n (%)		2 (15.4)	5 (29.4)		0.427
Family history of dementia, n (%)		5 (38.5)	1 (5.9)		0.061
Neuropsychological scores					
MMSE (/30)		21.85 ± 8.02	21.12 ± 9.15	0.228	0.822
MoCA (/30)		21.00 ± 5.71	19.27 ± 7.24	0.621	0.541

**Table 2:** Characteristics of APOE ε4 allele carriers and noncarriers.

	Genotype	AD patients (*n* = 14)		MCI patients (*n* = 16)	Total (*n* = 30)	*p* value
APOE4 carrier, APOE4+, n (%)	E2/E4 (n)	1	8 (61.5)	5 (38.5)	13	0.269
	E3/E4 (n)	10				
	E4/E4 (n)	2				
APOE4 noncarrier, APOE4−, n (%)	E3/E3 (n)	17	6 (35.3)	11 (64.7)	17	

### 3.2 Associations of regional cerebral blood flow with APOE4 allele

**Figure 1:**
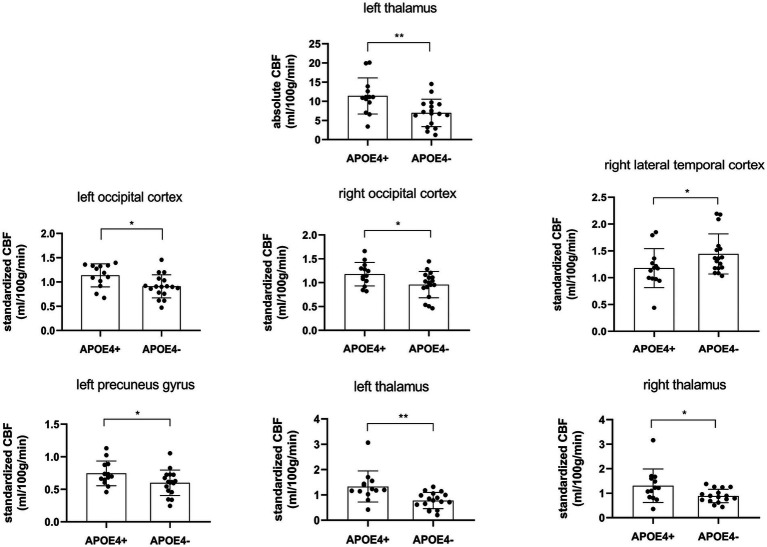
Regional cerebral blood flow was statistically different (*p* < 0.05) between APOE4 carriers and noncarriers. CBF, cerebral blood flow. *p < 0.05, **p < 0.01.

**Figure 2:**
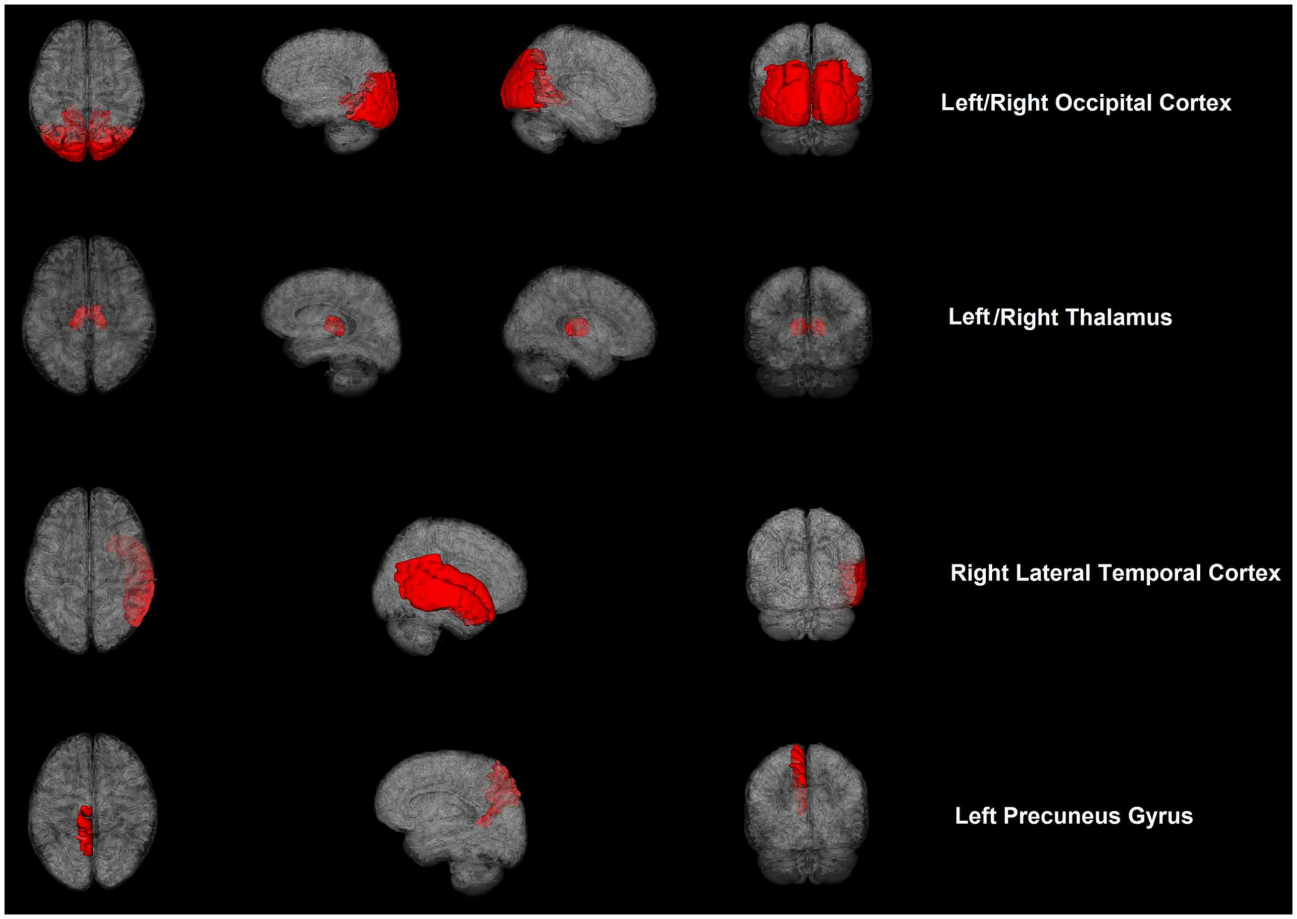
Cerebral blood flow maps of brain regions showed significant differences between APOE4 carriers and noncarriers with cognitive impairment.

rCBF exhibited significant differences between the two groups in some brain regions (Table 3; Figure 1). For absolute CBF, perfusion reduction was found in the APOE4− group in the left thalamus (*t* = 2.930, *p* = 0.007, difference value: 4.426, and 95% CI: 1.332–7.520). For standardized CBF, the APOE4− group demonstrated lower CBF than the APOE+ group in the bilateral occipital cortex (left: *t* = 2.568, *p* = 0.016, difference value: 0.226, and 95% CI: 0.046–0.406; right: *t* = 2.264, *p* = 0.032, difference value: 0.220, and 95% CI: 0.021–0.418), left precuneus gyrus (*Z* = −1.988, *p* = 0.047, difference value: 0.122, and 95% CI: 0.001–0.293) as well as bilateral thalamus (left: *Z* = −3.244, *p* = 0.001, difference value: 0.455, and 95% CI: 0.185–0.776; right: *Z* = −1.988, *p* = 0.047, difference value: 0.335, and 95% CI: 0.007–0.681). Additionally, the APOE4+ group exhibited hypo-perfusion in the right lateral temporal cortex (*Z* = 2.114, *p* = 0.035, difference value: 0.204, and 95% CI: 0.005–0.419). Spatially widespread CBF reduction was found between APOE4+ and APOE4− participants, which is illustrated in Figure 2.

**Table 3:** Differences in regional cerebral blood flow between APOE4 carriers and noncarriers.

Encephalic region	Group		t/Z value	*p* value	Difference values and 95% confidence intervals
	APOE4+ (*n* = 13)	APOE4− (*n* = 17)			
Absolute CBF					
Left thalamus	11.385 ± 4.709	6.959 ± 3.574	2.930	0.007	4.426 (1.332–7.520)
Standardized CBF					
Left occipital cortex	1.135 ± 0.238	0.910 ± 0.239	2.568	0.016	0.226 (0.046–0.406)
Right occipital cortex	1.177 ± 0.247	0.958 ± 0.275	2.264	0.032	0.220 (0.021–0.418)
Right lateral temporal cortex	1.173 (0.979, 1.315)	1.361 (1.177, 1.564)	−2.114	0.035	−0.204 (−0.419−0.005)
Left precuneus gyrus	0.698 (0.624, 0.882)	0.611 (0.468, 0.724)	−1.988	0.047	0.122 (0.001–0.293)
Left thalamus	1.198 (1.082, 1.494)	0.778 (0.504, 1.024)	−3.244	0.001	0.455 (0.185–0.776)
Right thalamus	1.213 (0.824, 1.635)	0.841 (0.705, 1.125)	−1.988	0.047	0.335 (0.007–0.681)

APOE4+, APOE4 carriers; APOE4−, APOE4 noncarriers.

### 3.3 Associations of bilirubin with APOE4 allele

**Figure 3:**
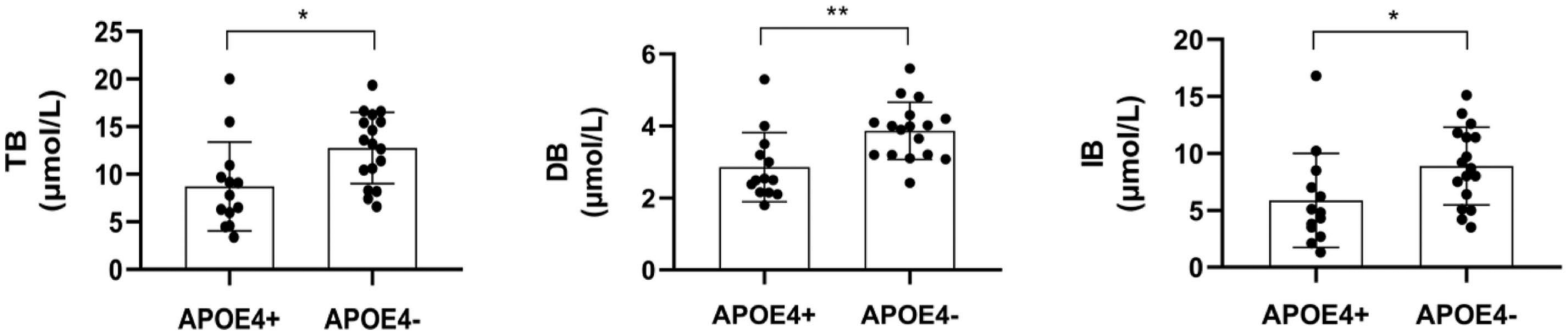
The comparison between serum liver function markers-bilirubin were statistically different (*p* < 0.05) between APOE4 carriers and noncarriers. TB, total bilirubin; DB, direct bilirubin; IB, indirect bilirubin. *p < 0.05, **p < 0.01.

We also investigated whether significant differences in serum liver function markers appeared in the contrast groups of APOE4+ versus APOE4−. The results indicated that there were significant differences in bilirubin levels between the two groups (Table 4; Figure 3). The APOE4+ group showed lower levels of bilirubin than the APOE4− group in TB (*Z* = 2.553, *p* = 0.011, difference value: 4.56, and 95% CI: 1.27–7.54), DB (*Z* = 2.973, *p* = 0.003, difference value: 1.06, and 95% CI: 0.56–1.72) and IB (*Z* = 2.366, *p* = 0.018, difference value: 3.50, and 95% CI: 0.70–6.30). However, there was no association between the two groups in terms of sundry protein and all liver enzymes (*p* > 0.05).

**Table 4:** Differences in bilirubin levels between APOE4 carriers and noncarriers.

Liver function marker	Group		Z value	*p* value	Difference values and 95% confidence intervals
	APOE4− (*n* = 17)	APOE4 + (*n* = 13)			
TB	13.20 (9.37, 15.89)	7.80 (5.28, 10.32)	2.553	0.011	4.56 (1.27–7.54)
DB	3.99 (3.20, 4.25)	2.50 (2.16, 3.35)	2.973	0.003	1.06 (0.56–1.72)
IB	8.70 (5.75, 11.60)	4.80 (3.10, 7.75)	2.366	0.018	3.50 (0.70–6.30)

APOE4+, APOE4 carriers; APOE4−, APOE4 noncarriers; TB, total bilirubin; DB, direct bilirubin; IB, indirect bilirubin.

## 4 Discussion

By exploring the associations of APOE4 with CBF and serum liver function markers in 30 cognitively impaired patients, two main findings were observed in the present study. First, when compared with noncarriers, the APOE4 carriers demonstrated significantly higher absolute CBF in the left thalamus; higher standardized CBF in the bilateral occipital cortex, bilateral thalamus, and left precuneus; and hypoperfusion in the right lateral temporal cortex. This indicates that APOE4 exerted a strong effect on the perfusion of certain brain regions. Second, APOE4 carriers manifested significantly lower levels of bilirubin (including TB, DB, and IB) than noncarriers. It is possible that an underlying mechanism of bilirubin metabolism is influenced by APOE4, which may hold promise as a target for the diagnosis and treatment of AD.

Previous work has established that APOE4 is associated with cerebral hemodynamic dysregulation (11, 16); however, recent studies have shown mixed observations, with evidence of hypoperfusion and hyperperfusion in APOE4 carriers showing cognitive decline (17). Michels et al. found that, between cognitively healthy older adults and patients with MCI, APOE4 carriers demonstrated lower CBF than noncarriers in frontal, parietal, and temporal areas (18). Intriguingly, another study reported that APOE4 carriers showed increased CBF in the right parahippocampal gyrus, bilateral cingulate gyrus, and right posterior cingulate in the MCI group, as well as the right superior frontal gyrus in the AD group (19). This result indicated that APOE4 carriers were also able to exhibit hyperperfusion in certain brain regions. Consistent with this, a recent study observed that middle-aged APOE4 carriers manifested increased CBF in the anterior and middle cerebral arteries (20). These studies demonstrate the biphasic nature of CBF in APOE4 carriers, and we hypothesize that different perfusion patterns correspond to different stages of cognitive decline.

Apart from discrepancies in sample characteristics, differences in perfusion imaging modalities, and post-processing approaches (20), cerebrovascular compensation also contributes to the conflicting observations. APOE4 carriers experience dysregulation of capillary blood flow with reduced oxygen transport to the brain tissue; consequently, increased CBF is needed to maintain sufficient oxygen supply and normal cognition (20, 21). Nevertheless, cerebrovascular compensation is limited with hyperperfusion in the early stage, resisting the deleterious impacts of APOE4 and subsequent hypoperfusion, indicating a relative breakdown of this compensatory mechanism (22, 23). Our findings help to indicate that the lateral temporal cortex may be one of the first brain regions to exhibit cerebrovascular compensation breakdown. In addition, we also provided evidence that APOE4 may be associated with CBF in the occipital cortex and thalamus, which has not been previously reported, and our findings also support that the thalamus is a vulnerable brain region in AD (24).

We also found that APOE4 carriers exhibited lower bilirubin levels than noncarriers. Bilirubin originates from the lysis of senescent erythrocytes and appears in the peripheral circulation in two forms: conjugated bilirubin (DB) and unconjugated bilirubin (IB). The primary form in the circulatory system is unconjugated bilirubin, which transforms into conjugated bilirubin by combining with albumin (ALB). Bilirubin participates in the decomposition and absorption of lipids (25) and its presence is used to diagnose conditions, such as hepatobiliary disorders and hemolytic anemia (26). Despite the lack of research on the relationship between APOE4 and bilirubin levels, some studies have provided relevant evidence. Zhong et al. reported that serum IB values and IB/ALB were significantly higher in patients with neurodegenerative dementia and Aβ deposition (27), indicating that bilirubin may be involved in the pathologic process of Aβ deposition. In contrast, higher serum bilirubin has been linked to increased regional homogeneity in certain brain regions within normal young adults (25), and AD patients have shown decreased concentrations of total bilirubin compared with normal subjects (28, 29), which supports our observations. Bilirubin performs powerful antioxidative and anti-inflammatory functions and can be considered a protective factor for scavenging superoxide during neurotransmission (25, 26). Although the specific mechanisms of APOE4 and bilirubin metabolism seem extremely complex, the above results imply that bilirubin could be a therapeutic target for cognitive impairment. Additionally, bile acid profiles produced by the liver were also linked to fluid and imaging biomarkers in patients with MCI and AD (30). Taken together, the liver may play a key role in the pathogenesis of cognitive impairment and its influence may be seriously underestimated.

The present study had several limitations. First, the sample size utilized in this study was relatively small, which may have compromised the reliability of the findings. Future studies should include larger cohorts of individuals diagnosed with AD and MCI, and the additional inclusion of normal controls would provide more substantial insights into the impact of APOE4 on brain perfusion and liver metabolic indicators. Second, as a cross-sectional design, the results were exploratory, and causal relationships could not be inferred from the analysis. Longitudinal research with interventions targeting cognitively impaired patients will help to identify a deeper understanding of the subject matter. Third, although ASL provides a noninvasive quantitative measurement of CBF and enhances the ability to detect cognitive decline and the progression of dementia (31–33), the ASL analysis results must still be interpreted carefully. The lack of harmonization of techniques complicates the analysis of data acquired from multiple scanners, and physiologic variations in human patients can cause strong variability in ASL acquired from the same patient at different time points, which may affect the confidence of the statistical analysis results in this study.

In conclusion, APOE4 inheritance exerts a strong impact on CBF dysfunction and represents a risk factor for AD. Different perfusion patterns may correspond to different stages of cognitive decline in APOE4 carriers. The lateral temporal cortex may be one of the first brain regions to show cerebrovascular compensation breakdown, and the thalamus may be a vulnerable brain region in AD. Further studies identifying APOE4−modulated CBF-specific mechanisms may provide options for the prevention and treatment of dementia via preclinical and clinical pathways. From a neurobiological perspective, APOE4 may be associated with bilirubin metabolism, as APOE4 carriers demonstrated lower bilirubin levels than noncarriers; this relationship could potentially provide specific neural targets for the diagnosis and treatment of AD.

## Data availability statement

The original contributions presented in the study are included in the article/Supplementary material, further inquiries can be directed to the corresponding author.

## Ethics statement

The studies involving humans were approved by Medical Research Ethics Committee of the Third Affiliated Hospital of Shenzhen University Medical College. The studies were conducted in accordance with the local legislation and institutional requirements. The participants provided their written informed consent to participate in this study.

## Author contributions

HW: Formal analysis, Writing – original draft, Writing – review & editing, Data curation, Investigation. LS: Formal analysis, Writing – review & editing. SL: Data curation, Writing – review & editing. YL: Formal analysis, Writing – review & editing. CX: Data curation, Writing – review & editing. GQ: Data curation, Writing – review & editing. QG: Data curation, Writing – review & editing. CC: Data curation, Writing – review & editing. TL: Formal analysis, Writing – review & editing. KL: Formal analysis, Writing – review & editing. FZ: Conceptualization, Formal analysis, Funding acquisition, Investigation, Methodology, Project administration, Resources, Supervision, Validation, Writing – review & editing.

## References

[1] Scheltens P de Strooper B Kivipelto M Holstege H Chételat G Teunissen CE. Alzheimer's disease. Lancet. (2021) 397:1577–90. doi: 10.1016/S0140-6736(20)32205-4, PMID: 33667416 PMC8354300

[2] Zheng W Cui B Han Y Song H Li K He Y. Disrupted regional cerebral blood flow, functional activity and connectivity in Alzheimer's disease: a combined ASL perfusion and resting state fMRI study. Front Neurosci. (2019) 13:738. doi: 10.3389/fnins.2019.00738, PMID: 31396033 PMC6668217

[3] Lou W Shi L Wong A Chu WC Mok VC Wang D. Changes of cerebral perfusion and functional brain network Organization in Patients with mild cognitive impairment. J Alzheimers Dis. (2016) 54:397–409. doi: 10.3233/JAD-160201, PMID: 27567823

[4] Miao G Zhuo D Han X Yao W Liu C Liu H. From degenerative disease to malignant tumors: insight to the function of ApoE. Biomed Pharmacother. (2023) 158:114127. doi: 10.1016/j.biopha.2022.114127, PMID: 36516696

[5] Farrer LA Cupples LA Haines JL Hyman B Kukull WA Mayeux R. Effects of age, sex, and ethnicity on the association between apolipoprotein E genotype and Alzheimer disease. A meta-analysis. APOE and Alzheimer disease Meta analysis consortium. JAMA. (1997) 278:1349–56. doi: 10.1001/jama.1997.03550160069041, PMID: 9343467

[6] Liu CC Zhao J Fu Y Inoue Y Ren Y Chen Y. Peripheral apoE4 enhances Alzheimer's pathology and impairs cognition by compromising cerebrovascular function. Nat Neurosci. (2022) 25:1020–33. doi: 10.1038/s41593-022-01127-0, PMID: 35915180 PMC10009873

[7] Hyman BT Gomez-Isla T Briggs M Chung H Nichols S Kohout F. Apolipoprotein E and cognitive change in an elderly population. Ann Neurol. (1996) 40:55–66. doi: 10.1002/ana.410400111, PMID: 8687193

[8] Reinvang I Espeseth T Westlye LT. APOE-related biomarker profiles in non-pathological aging and early phases of Alzheimer's disease. Neurosci Biobehav Rev. (2013) 37:1322–35. doi: 10.1016/j.neubiorev.2013.05.006, PMID: 23701948

[9] Liu CC Liu CC Kanekiyo T Xu H Bu G. Apolipoprotein E and Alzheimer disease: risk, mechanisms and therapy. Nat Rev Neurol. (2013) 9:106–18. doi: 10.1038/nrneurol.2012.263, PMID: 23296339 PMC3726719

[10] Lane-Donovan C Wong WM Durakoglugil MS Wasser CR Jiang S Xian X. Genetic restoration of plasma ApoE improves cognition and partially restores synaptic defects in ApoE-deficient mice. J Neurosci. (2016) 36:10141–50. doi: 10.1523/JNEUROSCI.1054-16.2016, PMID: 27683909 PMC5039258

[11] Tai LM Thomas R Marottoli FM Koster KP Kanekiyo T Morris AW. The role of APOE in cerebrovascular dysfunction. Acta Neuropathol. (2016) 131:709–23. doi: 10.1007/s00401-016-1547-z, PMID: 26884068 PMC4837016

[12] Martínez-Morillo E Hansson O Atagi Y Bu G Minthon L Diamandis EP. Total apolipoprotein E levels and specific isoform composition in cerebrospinal fluid and plasma from Alzheimer's disease patients and controls. Acta Neuropathol. (2014) 127:633–43. doi: 10.1007/s00401-014-1266-2, PMID: 24633805

[13] Jack CR Jr Bennett DA Blennow K Carrillo MC Dunn B Haeberlein SB. NIA-AA research framework: toward a biological definition of Alzheimer's disease. Alzheimers Dement. (2018) 14:535–62. doi: 10.1016/j.jalz.2018.02.018, PMID: 29653606 PMC5958625

[14] Albert MS DeKosky ST Dickson D Dubois B Feldman HH Fox NC. The diagnosis of mild cognitive impairment due to Alzheimer's disease: recommendations from the National Institute on Aging-Alzheimer's Association workgroups on diagnostic guidelines for Alzheimer's disease. Alzheimers Dement. (2011) 7:270–9. doi: 10.1016/j.jalz.2011.03.008, PMID: 21514249 PMC3312027

[15] Tzourio-Mazoyer N Landeau B Papathanassiou D Crivello F Etard O Delcroix N. Automated anatomical labeling of activations in SPM using a macroscopic anatomical parcellation of the MNI MRI single-subject brain. NeuroImage. (2002) 15:273–89. doi: 10.1006/nimg.2001.0978, PMID: 11771995

[16] Wisniewski T Drummond E. APOE-amyloid interaction: therapeutic targets. Neurobiol Dis. (2020) 138:104784. doi: 10.1016/j.nbd.2020.104784, PMID: 32027932 PMC7118587

[17] Luckhaus C Cohnen M Flüβ MO Jänner M Grass-Kapanke B Teipel SJ. The relation of regional cerebral perfusion and atrophy in mild cognitive impairment (MCI) and early Alzheimer's dementia. Psychiatry Res. (2010) 183:44–51. doi: 10.1016/j.pscychresns.2010.04.003, PMID: 20541374

[18] Michels L Warnock G Buck A Macauda G Leh SE Kaelin AM. Arterial spin labeling imaging reveals widespread and Aβ-independent reductions in cerebral blood flow in elderly apolipoprotein epsilon-4 carriers. J Cereb Blood Flow Metab. (2016) 36:581–95. doi: 10.1177/0271678X15605847, PMID: 26661143 PMC4794091

[19] Kim SM Kim MJ Rhee HY Ryu CW Kim EJ Petersen ET. Regional cerebral perfusion in patients with Alzheimer's disease and mild cognitive impairment: effect of APOE epsilon4 allele. Neuroradiology. (2013) 55:25–34. doi: 10.1007/s00234-012-1077-x, PMID: 22828738

[20] Dounavi ME Low A McKiernan EF Mak E Muniz-Terrera G Ritchie K. Evidence of cerebral hemodynamic dysregulation in middle-aged APOE ε4 carriers: the PREVENT-dementia study. J Cereb Blood Flow Metab. (2021) 41:2844–55. doi: 10.1177/0271678X211020863, PMID: 34078163 PMC8543665

[21] Østergaard L Aamand R Gutiérrez-Jiménez E Ho YC Blicher JU Madsen SM. The capillary dysfunction hypothesis of Alzheimer's disease. Neurobiol Aging. (2013) 34:1018–31. doi: 10.1016/j.neurobiolaging.2012.09.011, PMID: 23084084

[22] Hays CC Zlatar ZZ Wierenga CE. The utility of cerebral blood flow as a biomarker of preclinical Alzheimer's disease. Cell Mol Neurobiol. (2016) 36:167–79. doi: 10.1007/s10571-015-0261-z, PMID: 26898552 PMC5278904

[23] Koizumi K Hattori Y Ahn SJ Buendia I Ciacciarelli A Uekawa K. Apoε4 disrupts neurovascular regulation and undermines white matter integrity and cognitive function. Nat Commun. (2018) 9:3816. doi: 10.1038/s41467-018-06301-2, PMID: 30232327 PMC6145902

[24] Aggleton JP Pralus A Nelson AJ Hornberger M. Thalamic pathology and memory loss in early Alzheimer's disease: moving the focus from the medial temporal lobe to Papez circuit. Brain. (2016) 139:1877–90. doi: 10.1093/brain/aww083, PMID: 27190025 PMC4939698

[25] Chen J Liu S Wang C Zhang C Cai H Zhang M. Associations of serum liver function markers with brain structure, function, and perfusion in healthy young adults. Front Neurol. (2021) 12:606094. doi: 10.3389/fneur.2021.606094, PMID: 33716920 PMC7947675

[26] Vasavda C Kothari R Malla AP Tokhunts R Lin A Ji M. Bilirubin links Heme metabolism to neuroprotection by scavenging superoxide. Cell Chem Biol. (2019) 26:1450–1460.e7. doi: 10.1016/j.chembiol.2019.07.006, PMID: 31353321 PMC6893848

[27] Zhong X Liao Y Chen X Mai N Ouyang C Chen B. Abnormal serum bilirubin/albumin concentrations in dementia patients with Aβ deposition and the benefit of intravenous albumin infusion for Alzheimer's disease treatment. Front Neurosci. (2020) 14:859. doi: 10.3389/fnins.2020.00859, PMID: 33013289 PMC7494757

[28] Hatanaka H Hanyu H Fukasawa R Hirao K Shimizu S Kanetaka H. Differences in peripheral oxidative stress markers in Alzheimer's disease, vascular dementia and mixed dementia patients. Geriatr Gerontol Int. (2015) 15:53–8. doi: 10.1111/ggi.12659, PMID: 26671158

[29] Vasantharekha R Priyanka HP Swarnalingam T Srinivasan AV ThyagaRajan S. Interrelationship between Mini-mental state examination scores and biochemical parameters in patients with mild cognitive impairment and Alzheimer's disease. Geriatr Gerontol Int. (2017) 17:1737–45. doi: 10.1111/ggi.12957, PMID: 27921357

[30] Nho K Kueider-Paisley A MahmoudianDehkordi S Arnold M Risacher SL Louie G. Altered bile acid profile in mild cognitive impairment and Alzheimer's disease: relationship to neuroimaging and CSF biomarkers. Alzheimers Dement. (2019) 15:232–44. doi: 10.1016/j.jalz.2018.08.012, PMID: 30337152 PMC6454538

[31] Wierenga CE Hays CC Zlatar ZZ. Cerebral blood flow measured by arterial spin labeling MRI as a preclinical marker of Alzheimer's disease. J Alzheimers Dis. (2014) 42:S411–9. doi: 10.3233/JAD-141467 25159672 PMC5279221

[32] Camargo A Wang Z. Alzheimer’s Disease Neuroimaging Initiative. Longitudinal cerebral blood flow changes in Normal aging and the Alzheimer's disease continuum identified by arterial spin labeling MRI. J Alzheimers Dis. (2021) 81:1727–35. doi: 10.3233/JAD-210116, PMID: 33967053 PMC8217256

[33] Chao LL Buckley ST Kornak J Schuff N Madison C Yaffe K. ASL perfusion MRI predicts cognitive decline and conversion from MCI to dementia. Alzheimer Dis Assoc Disord. (2010) 24:19–27. doi: 10.1097/WAD.0b013e3181b4f736, PMID: 20220321 PMC2865220

